# North-Eastern Hospital for Children

**Published:** 1902-05-31

**Authors:** 


### NORTH-EASTERN HOSPITAL FOR CHILDREN.

### A NEW ERA.

THE visitors of King Edward's Fund, who made an inspection just before the annual meeting on Friday, the 23rd inst., were reported to have said, "It's a nice little hospital, but you want more room."

The remarks of the Chairman, Lieut.-Colonel Lord William Cecil, in presenting the annual report and accounts, were also very much to the point—"All satisfactory so far as it goes." How far it will go in the future remains to be seen, but at any rate a great step forward was taken when, rather more than a fortnight ago, H.R.H. Princess Henry of Battenberg laid the memorial stone of the new wing.

The motions put were all carried without discussion; and the Chairman announced, amid applause, that H.R.H. Princess Henry of Battenberg had consented to become a patron. The Duke and Duchess of Somerset were also elected patrons, and the Lord Bishop of London was made a Vice-President.

Mr. Abram Lyle said he thought a new era had dawned for the hospital, now that so many kind friends were coming forward to give their support.

The office of President is still vacant; the Chairman thought it would be difficult to find anyone not already engaged in similar work; and he added that the selection should be made with great care.

Mr. Charles G. Port proposed the following resolution:—

"That the committee be authorised to arrange for an advance of any sum not exceeding £12,000, to be secured by a mortgage of the property of the hospital, to enable them to pay for the new buildings now in progress, and that the trustees be authorised to execute the necessary deed or deeds to carry the loan into effect."

Mr. Port said the governors were probably aware that when the building was begun there was not money in hand to cover the cost. Operations were delayed for two years in order that the committee might feel that they were justified in incurring an expenditure of about £30,000. When something like half of this was in hand, they felt that a beginning might be made. Though chairman of the finance committee, he had no more to do with the money than any other member, but he wished to point out that in all probability it would be necessary to borrow a certain amount. The committee did not think they would have to borrow £12,000, but they would have to borrow something. The building would entail increased yearly expenditure, but that, he hoped, would be met by the ordinary income. The payment of the balance on the building fund must be met by extraordinary efforts. The motion was seconded by Mr. Meller, and carried unanimously.

The next business was a motion by Mr. Francis Howse:—

"That the trustees be authorised to effect an exchange with Mr. Archibald Edward Scott of two small portions of land belonging to the hospital in the rear of the former site of No. 337 Hackney Road, for two small pieces of land belonging to the said Mr. Archibald Edward Scott, the adjoining owner, for the purpose of rectifying part of the eastern boundary of the hospital property."

Mr. Howse said the hospital would have to pay £50 and Mr. Scott's costs. It had been found necessary to ask the consent of the governors before the deed of transfer could be signed. In reply to the question of a governor, Mr. Howse said Mr. Scott had been approached, but that he would have not only the exchange but the money and expenses as well.

The motion was seconded by the Mayor of Hackney, Mr. Walter Johnson, J.P., and carried unanimously.

The Chairman said he would like to suggest that a letter of thanks should be sent to Lord Frederick FitzRoy, the retiring chairman; and Miss Phillips, the foundress of the hospital, said that Lord FitzRoy used to come down before there was any committee at all; indeed he had once helped to lay the table when the hospital was up in Hackney Road. She looked back to his kindness with very great gratitude.

About seventeen governors were present, three of whom were ladies.

